# King Edward VII.'s Hospital for Officers

**Published:** 1904-04-23

**Authors:** 


### The Hospital.

A Journal of

The Medical Sciences and Hospital Administration.

Vol. XXXVI.—No. 917. APRIL 23, 1904.

### King Edward VII.'s Hospital for Officers.

It is a matter of common knowledge that the warm interest taken by his Majesty the King in the naval and military services is by no means limited to those questions of efficiency which are of the highest importance to the State, but extends also to everything which can be conducive to the comfort and welfare of individuals. Of this there could be no better illustration than has been furnished by the share taken by his Majesty in the provision and equipment of the London hospital for officers of both services, which has just been opened at 9 Grosvenor Gardens, mainly by the exertions, and under the control, of the patriotic and benevolent lady who prefers to be known to the public only as "Sister Agnes," but whose work on behalf of sick and wounded officers, both during the whole of the South African war and subsequently, has been sufficient to show that the spirit which animated Miss Nightingale is still active in our midst, and that she, like Ridley and Latimer in Smithfield, lighted a torch in England which will never be suffered to go out. Officers of both services have for a long time enjoyed the privilege of hospital treatment, at Haslar or at Netley, in circumstances requiring it, and to this privilege the generosity of his Majesty has lately added that of admission to the admirable convalescent hospital which he has established at Osborne House; but it was reserved for Sister Agnes to point out the still unsupplied want of a hospital in London, in the immediate vicinity not only of the heads of the medical and surgical professions, but also of the naval and military authorities, and easily accessible to the friends and relatives of patients from every part of the country. Having in the first place submitted her proposal to the King, and having secured his Majesty's cordial approval and support, Sister Agnes at once proceeded to forge what had previously been a missing link in the general arrangements for the treatment of sick or wounded officers; and, largely from her own resources, although with assistance from friends, secured and equipped the house in Grosvenor Gardens, which was lately the town residence of Lord Churchill, and opened it on Monday last as the London branch, so to speak, of Osborne House, for the reception of 12 cases of serious character, or requiring treatment of a kind for which the metropolis affords special facilities. The patients admitted will make only the moderate payments which, by the regulations of the services, would be required from them at Haslar or at Netley; but the maintenance of the establishment is for the present provided for by friends, each of whom has promised Sister Agnes an annual contribution of £100 for five years certain, a guarantee under which she has consented to undertake all the responsibilities of the establishment and to conduct it personally for the specified time. The list of these contributors is headed by his Majesty and by the Prince of Wales, and it can hardly be doubted, when the experience of five years has shown the value and utility of the institution, that its future will be secured by the liberality of the services themselves, even if only as a method of insurance against the consequences of injury or disease.

Established under these favourable conditions, the equipment of the hospital leaves nothing to be desired, and is equally calculated to satisfy the requirements of the scientific and the tastes of the fastidious. The experience of Sister Agnes has given her a complete knowledge of the essentials of nursing, while her education and social position render her equally conversant with the needs of culture. She has wisely determined to be guided in all respects rather by her own knowledge than by the advice of others, however well meaning; and the result is to be seen in a sort of unity of completeness, difficult to describe but easy to appreciate, which pervades the hospital as a whole. The operating theatre, where the highest attainable degree of safety has been secured, seems to afford no possible lurking place for even the most modest and most retiring of microbes; and it contains everything by which the work of the surgeon can be facilitated, such as admirable lighting, both natural and artificial, easy means of regulating temperature, and all needful appliances for the sterilisation of hands, dressings, and instruments. The wards, of which there are seven, five containing two beds each, and two smaller ones that are single-bedded, present a delightful combination of prettiness with simplicity; but Sister Agnes calls special attention to the beauty of her counterpanes, on which an exquisite pattern forms a border for the Royal monogram. The ward furniture is of wood, enamelled white, and has no angles or crannies to afford lodgment to dust, while the walls are washable. As the visitor passes away from them, and enters the day-rooms, every trace of association with sickness disappears, unless it be the presence of a slight inclined plane, over which a chair may be wheeled from the passage into the billiard room without hindrance from an intervening step. The front drawing-room is simply a luxuriously furnished sitting-room, rather suggestive of a club, and containing a sufficiency of easy chairs, couches, and such-like aids to comfort, besides books in abundance, writing materials, and telephones by which it may be brought into relation with public performances, or which may equally be subservient to private uses. The back drawing-room contains an excellent billiard table, and there is a cosy little dining-room for patients who are sufficiently well to be able to resort to it. We cordially congratulate the officers of both services on this new and altogether splendid provision for their needs, and venture to predict for the new hospital a long and brilliant career of usefulness.

